# Technology Roadmaps for Compound Semiconductors

**DOI:** 10.6028/jres.105.036

**Published:** 2000-06-01

**Authors:** Herbert S. Bennett

**Affiliations:** National Institute of Standards and Technology, Gaithersburg, MD 20899-8120

**Keywords:** analog to digital converters, compound semiconductors, digital video systems, technology roadmaps, wireless communications

## Abstract

The roles cited for compound semiconductors in public versions of existing technology roadmaps from the National Electronics Manufacturing Initiative, Inc., Optoelectronics Industry Development Association, Microelectronics Advanced Research Initiative on Optoelectronic Interconnects, and Optoelectronics Industry and Technology Development Association (OITDA) are discussed and compared within the context of trends in the Si CMOS industry. In particular, the extent to which these technology roadmaps treat compound semiconductors at the materials processing and device levels will be presented for specific applications. For example, OITDA’s Optical Communications Technology Roadmap directly connects the information demand of delivering 100 Mbit/s to the home to the requirement of producing 200 GHz heterojunction bipolar transistors with 30 nm bases and InP high electron mobility transistors with 100 nm gates. Some general actions for progress towards the proposed International Technology Roadmap for Compound Semiconductors (ITRCS) and methods for determining the value of an ITRCS will be suggested. But, in the final analysis, the value added by an ITRCS will depend on how industry leaders respond. The technical challenges and economic opportunities of delivering high quality digital video to consumers provide concrete examples of where the above actions and methods could be applied.

## 1. Introduction and Motivation

During the last 5 or so years, a compound semiconductor industry that is distinct from the silicon semiconductor industry has emerged. Unlike the silicon industry, the compound semiconductor industry does not have an international consensus for a few selected applications and markets to set priorities for investments [1]. The main purpose of this paper is to increase the awareness among industrial decision-makers about the need for an international consensus concerning compound semiconductors [2]. In addition, other purposes of this paper are to:

1. Present general principles and guidelines for undertaking an International Technology Roadmap for Compound Semiconductors (ITRCS).
2. Identify candidate technology challenges in compound semiconductors for a few specific systems that will serve to focus some of the general principles presented here. It is important that this focus contain enough cross-cutting technologies to be representative of the compound semiconductor manufacturing infrastructure and yet specific enough for success. Wireless broadband and high-speed digital communications networks, especially those for digital video, are prime applications and markets on which an ITRCS could focus.
3. Encourage comments from others.

Technology roadmaps are an effective technique to reduce uncertainties in investments. They are not an end, but a beginning. When viewed retrospectively, the technologies considered in a given edition of a roadmap may develop differently than first thought. Technology roadmaps by themselves are useful documents of what the individuals who generated the roadmaps discussed, concluded at a given time, and decided to put into a retrievable format for the written record. However, the greater value of roadmaps lies not in the written record, but in bringing about industrial cooperation and changes in how companies work together. Often, the individuals who contribute to technology roadmaps realize in due course that more than half of what they know may be treated as non-proprietary information and shared in a cooperative manner with other companies. Successful roadmaps like the International Technology Roadmap for Semiconductors (ITRS) [3] required changes in the attitude and culture among those companies that participate for the common well being of their industry. Roadmaps become more successful when attitudes and cultures change among decision-makers in companies so that they may cooperate more effectively. Then, they will be able to share concerns, technical challenges, and priorities, and to establish common technical goals and a more robust infrastructure for their industry. That is perhaps the most significant lesson learned by the Si CMOS industry during the past two decades. The very existence of roadmaps that mention compound semiconductors means that decision-makers in the compound semiconductor industry have started to alter their attitudes thereby enabling more cooperation. However, much more remains to be accomplished in order to approach the benchmark established by the Si CMOS industry.

## 2. Trends in the Silicon CMOS Industry

The competitiveness among silicon CMOS manufacturers is shifting from an emphasis on technology and fabrication to a much greater emphasis on product design, architecture, algorithm, and software; that is, it is shifting from technology oriented R&D to product-oriented R&D. Other trends include:

1. Increased costs for R&D and production facilities are becoming too great for any one company or country to accept.
2. Shorter process technology life cycles.
3. Emphasis on faster characterization of manufacturing processes.
4. All-global participants in the “Si CMOS ecosystem” now collaborate to develop and improve manufacturing technologies; for example, ITRS and International SEMATECH.
5. Many observers credit collaborative, consensus-based planning and deliberate road-mapping efforts for the sustained average annual growth rate of 15 % for the Si CMOS semiconductor industry over this past decade.

One of the major themes of the 1998 International Workshop on Future Trends in Microelectronics: The Road Ahead, [4] was that there are limits to the continued growth of the Si CMOS industry even on an international scale. We have already seen, for example, by the formation in April 1998 of an International SEMATECH, that individual nations do not have adequate resources for the next generation of Si CMOS and that international collaboration will be required for 300 mm wafers. Bringing 300 mm wafer fabrication facilities on-line was slowed due to a combination of technical and economic factors. Also, the 1999 ITRS has data showing the design productivity gap; that is, the number of transistors per chip is increasing at about 58 % per year, but the number of transistors per designer-month is increasing only at about 21 % per year [5]. These observations and many of the other comments made during this Workshop are consistent with the research of Derek de Solla Price [6].

It is worthwhile to consider Price’s earlier results in the context of today’s road-mapping efforts. In his 1971 talk [6], Prof. Price summarized his research based on market analyses, interviews of leading technologists, and examinations of patents, archival publications, and citations for patents and publications. Commercial technologies and markets have three main phases of development. These are growth, saturation, and sometimes diminishing markets. The factors that determine the saturation phase include:

1. Finite extent of any economy: The required resources to continue the advancement of a given technology, such as Si CMOS, cannot exceed the available money supply.
2. Manpower limit: One technology, such as Si CMOS, cannot capture most of the available human resources in order to work on advancing that technology.

Based on his research data, Price formulated relationships that are still relevant to portions of the microelectronics industry. During the growth phase, he discerned such relationships as the following:

1. High quality technology development grows at a slower rate than low quality technology development.
2. The number of high quality developments is proportional to the *n*th root of the total number of developments, where *n* is greater than 2 or 3 and depends on the technical field.
3. Resources and funds devoted to a given technology are proportional to the nth power of the number of people working on that technology. This implies technology growth will be limited eventually by a lack of resources, both financial and human.
4. Doubling the size of a technological effort does not double the amount of useful results. His data suggest that the useful results vary as the *m*th root of the size of the effort, where *m* is between 2 and 4 for most technologies.

According to Price, technology deployment, unlike science, is more like the arts and may be localized as language is localized. For the Si CMOS industry, this last statement becomes equivalent to the statement that the ITRS gives international needs and it remains for local domestic decision makers to select those needs for which local resources will be used to provide solutions. This is just what the new International SEMATECH and SRC will be doing.

## 3. Trends in the Compound Semiconductor Industry

A similar shift from technology-based R&D to product-based R&D may occur for one or two major applications of compound semiconductors, particularly, in applications for which III-V compound semiconductors and elemental silicon or silicon-germanium semiconductors co-exist. The issue is not so much compound semiconductors versus elemental silicon, but compound semiconductor processes that are not compatible with Si CMOS versus compound semiconductor processes such as SiGe bipolar and SiGe tunnel diodes that are compatible with Si CMOS.

The expected trends in compound semiconductors, particularly those whose processing is not compatible with silicon CMOS processing, suggest that the infrastructure for consensus-based planning needs to be strengthened. In general, the resources available to those companies involved with compound semiconductors are much smaller than the resources available to those companies involved with mainstream silicon CMOS. Coordinated planning is one way to invest better the limited resources for compound semiconductor technologies. This is especially the case for those technologies that depend in part on III-V compound semiconductors such as GaAs. During the last 5 years, some companies, which produced primarily III-V compound semiconductors for commercial wireless systems operating at frequencies less than a few GHz, have added Si and SiGe processes. They may be moving away from III-V compound semiconductors operating at these lower frequencies in favor of element IV compounds such as BiCMOS and SiGe that are much more compatible with mainstream Si CMOS processing [7]. We do not know whether today’s profits made from lower-frequency III-V technologies, which are likely to be lost to the aggressive Si and SiGe technologies, will be comparable to or greater than the future profits made from higher-frequency III-V technologies, which operate at tens of GHz and for which Si and SiGe technologies do not have adequate performance. Many believe that III-V compound semiconductors will enable emerging markets such as millimeter wave communications and local multipoint distribution service (LMDS) and that the profits from these new markets will exceed the profits lost to Si CMOS compatible technologies. Another view is that because the wireless markets below about 6 GHz are growing so fast today, the III-V compound semiconductor industry may share those markets with Si CMOS processes and still have increases in profits.

SiGe-base bipolar transistors are making rapid inroads into high-frequency small-signal and analog applications and the III-V compound semiconductor industry needs to know the extent to which they will permanently displace GaAs or other compound semiconductor devices such as heterojunction bipolar transistors (HBTs). In his recent talk [8], T. Ning compared GaAs HBTs and SiGe HBTs. When GaAs HBTs and SiGe HBTs are made with the same design rules and from similar advanced structures, and most importantly, when they are measured at the same collector current density, GaAs HBTs are inherently faster, less noisy, and much more scaleable than SiGe bipolar transistors. GaAs HBTs also have lower impact ionization rates. Ning further showed that most SiGe bipolar transistors that have been reported as HBTs are not really heterojunction bipolar transistors at all. The inherent advantage of the SiGe bipolar transistor over the GaAs HBT lies in its being compatible with Si CMOS processing.

This last trend of competition from elemental and group IV compound semiconductors places a greater urgency for coordinated, consensus-based planning concerning those compound semiconductors, perhaps primarily III-V semiconductors, that are not compatible with Si CMOS processes. The greatest economic challenge facing the III-V compound semiconductor industry is to increase its volume and decrease costs dramatically for high quality and advanced performance III-V compound semiconductor devices and circuits.

## 4. International Technology Roadmap for Compound Semiconductors

### 4.1 Applying the ITRS Model to Compound Semiconductors

Because of limited resources in any one nation or economic region, the proposed planning for compound semiconductors should be international in scope and involve at the least companies and universities from Asia, Europe, North America, and Australia.

Suggesting from the start that planning for compound semiconductors should be an international effort is consistent with the silicon CMOS efforts at SEMATECH/International SEMATECH, Semiconductor Industry Association (SIA), and Semiconductor Research Corporation (SRC). The formation of International SEMATECH, a subsidiary of SEMATECH, was announced on April 2, 1998 [9]. International SEMATECH has programs on lithography infrastructure, standards, and environmental health and safety. At the beginning of January 2000, SEMATECH and International SEMATECH merged into one entity called International SEMATECH. Earlier, the SIA, SEMATECH, and International SEMATECH decided to internationalize the process for creating the 1999 version of the Technology Roadmap for Semiconductors, now called the International Technology Roadmap for Semiconductors (ITRS) [3]. The focus of the ITRS will be on needs of the Si CMOS industry. Participating organizations will be able to develop internal domestic versions and address potential solutions to selected ITRS needs, as they may desire. In the last half of 1999, the SRC opened membership to companies worldwide. L. Sumney, SRC’s president and CEO, said in a recent press release [3] “Just as chips have gotten smaller, the world has gotten smaller. Isolated research will not solve today’s technical challenges; global cooperation and sharing of information will.”

Following the global trends in the Si CMOS industry, we similarly propose here that the international planning for compound semiconductors emphasize technical barriers and needs, and that participating organizations be free to develop internal domestic plans to provide solutions.

### 4.2 Motivation and Focus

Because compound semiconductor technologies are very diverse, a focus is needed. It has been suggested that wireless, real-time digital video could be a strong candidate for providing this focus. Section 10 contains a discussion of some technical challenges associated with delivering wireless, high quality digital video to consumers. Any credible plan for wireless digital video would have to consider compound semiconductor technologies for such applications as front ends of receivers, analog-to-digital converters, equalizers, error correction circuits, and optoelectronic integrated circuits.

Some have stated that motivating a compound semiconductor technology roadmap is difficult. They believe that, unlike the case for Si CMOS, an equivalent to Moore’s Law does not exist and market shares have not been altered sufficiently among competitors to induce one subset of competitors to undertake a cooperative technology roadmap analogous to the ITRS. But, III-V compound semiconductor field effect transistors (FETs) and analog-to-digital converters (ADC) are in fact constrained by the density of devices. Also, even though many applications for compound semiconductors are not constrained by the increase in the density of devices as a function of time (as described by Moore’s Law), equivalents to Moore’s Law for figures of merit such as gain and operating frequency of transceiver front ends do exist. Later in this paper, data are presented which show that a definite “Moore’s Law” for the increase of ADC figures of merit with time exists. However, instead of doubling every 18 months as is the case for Si CMOS, the bit resolution of ADCs increases between 1 bit and 2 bits at the same sampling rate every 8 years or so.

At the other end of the spectrum, the key trade-offs between III-V compound semiconductors and Si semiconductors are the questions of availability of appropriate benchmarking standards so that design engineers and manufacturers may communicate with one another to meet production schedules. Some of these benchmarking standards include performance parameters such as gain, efficiency, noise, linearity, power consumption, and thermal management. Most III-V compound semiconductor devices have higher gains with higher operating and maximum frequencies. But, they often have lower thermal conductivities. The latter is often at the hub of the technical debate on GaAs/AlGaAs heterojunction bipolar transistors (HBTs) versus Si or SiGe bipolar transistors. Providing credible graphs on how these quantities vary with the time is not straightforward because agreed-upon, high-quality ways to measure these quantities at the highest frequencies are not readily available. This is one example that demonstrates the requirement for improved measurement capabilies to be included in technology roadmaps.

The market share that GaAs/AlGaAs HBTs now have for 1.8 GHz to 2.5 GHz wireless applications is being challenged by Si BiCMOS [10,11] and SiGe technologies. The rf applications for BiCMOS in Ref. [10] may become the next example that illustrates how Si CMOS and Si BiCMOS are aggressive technologies that migrate into markets previously dominated by compound semiconductors. This situation is analogous in some aspects to the market share shift that occurred among competing Si CMOS companies and that led after many years to the NTRS. In the case of III-V compound semiconductors, market share losses to Si-based technologies and to processes that are more compatible with Si CMOS processing are occuring at the moving boundary between III-V and Si technologies. The application of microelectronic devices to mobile and very high bit rate communication systems operating from 900 MHz to 6 GHz is a good candidate for consensus-based planning. High bit rate, mobile communications are mass market drivers for which performance and cost are very important and encompass many Si, SiGe, and III-V compound semiconductor devices and circuits.

Even though individual companies and associations have their own compound semiconductor plans, a more comprehensive and globally based plan for one or two applications of compound semiconductors with potentially large markets does not exist. Today, III-V compound semiconductor companies compete on technology, fabrication, and design. This mode of competition among silicon CMOS manufacturers is changing because the research and development costs associated with advanced larger wafer sizes and smaller linewidths for CMOS are too great for any one company or country to accept [12]. The competitiveness among CMOS manufacturers is shifting from technology and fabrication to product design, supported substantially by advanced computer simulations.

## 5. ITRS—The Benchmark Roadmap

We first consider the ITRS’s December 1999 Report [3] as a benchmark for the other technology roadmaps that will be briefly reviewed in the following Section. The ITRS is very material- and process-specific because it is limited primarily to crystalline Si CMOS for the three very big market applications of microprocessor-logic circuits, memories, and mixed signal circuits.

It contains reasonably simple metrics for determining progress (for example, line width and density of devices in circuits). The ITRS has two very unique features that are not presented with nearly as much detail in other technology roadmaps:

1. The ITRS presents its interim yearly target goals in terms of acceptable tolerances and variations in device- and circuit-figures of merits, and relates such tolerances and variations to required materials processing controls on key parameters such as linewidth, doping density, and layer thickness.
2. The ITRS identifies the relatively near-term targets that can be met by known technology solutions currently under development and other longer-term technology targets for which there are no known reliable approaches to continued scaling of CMOS technology and processes. If solutions are not found or are likely to be developed later, then progress might end unless some real breakthroughs are achieved.

## 6. Existing Roadmaps—Compound Semiconductors

Segments of the III-V compound semiconductor industry have contributed to some technology roadmaps that deal, in part, with selected challenges for their industry. However, these roadmaps tend to be fragmented efforts as far as compound semiconductors are concerned and lack the details for infrastructure building that would be comparable to those in the ITRS for Si CMOS. From the historical perspective, the ongoing road-mapping activities in North America (NEMI [13] and OIDA [14]), Europe (MEL-ARI-OPTO [15]), and Japan (OITDA [16]) may signify, as did the early planning efforts by SIA, SRC, MICROTECH 2000, and SEMATECH for silicon, that the time is ripe for a more global road-mapping initiative concerning compound semiconductors.^1^

Using the ITRS as a benchmark for comparison and keeping the discussion in the last paragraph of Sec. 1 in mind, we now review briefly each of the recent and publicly available technology roadmaps from NEMI, OIDA, MEL-ARI-OPTO, and OITDA. Although all four of these roadmaps mention III-V compound semiconductors, they do not have well-developed plans with features analogous to those in the previous Section on the ITRS (items 1 and 2).

Roadmaps from the National Electronics Manufacturing Initiative (NEMI) December 1998 Report [13] treat numerous market applications and have some specifically compound semiconductor-related content in the areas of energy storage, radio frequency devices, and optoelectronic integrated circuits and components; see http://www.nemi.org/.

Roadmaps from the Optoelectronics Industry Development Association (OIDA) October 1996 Report [14] treat several diverse market applications and have some relevance to compound semiconductors in the areas of sensors, detectors, and displays.

The Microelectronics Advanced Research Initiative Optoelectronics Technology Roadmap (MEL-ARI OPTO) [15] is a European Commission effort in the area of III-V semiconductor interconnects for integrated circuits. Its June 1998 Report contains discussions of specific III-V compounds for optoelectronic interconnects and drivers, and detector/receiver arrays used in numerous market applications.

The roadmap from the Optoelectronics Industry and Technology Development Association (OITDA), August 1998 Report [16], has sections that treat specific materials for use in light sources, optical modulators and receivers, and ultra-high-speed electronic devices. These have applications in numerous markets. As an example of material specificity, this OITDA Roadmap directly connects the information demand of 100 Mbit/s per home to the requirement of producing 200 GHz III-V HBTs with 30 nm bases and InP HEMTs with 100 nm gates.

## 7. Lessons Learned From ITRS and NEMI

Reviewing the history of the ITRS and the NEMI Roadmaps is very worthwhile for those building an international consensus concerning III-V compound semiconductors. The history of the ITRS offers several lessons that are applicable to the compound semiconductor industry. These lessons include:

1. Prior to the mid 1980s, most Si CMOS companies assumed that over 50 % of what they knew was proprietary and not to be part of consensus-based planning and collaborations.
2. From the late 1980s to today, most Si CMOS companies found that over 50 % of what they know is not proprietary and may be shared with other companies for a globally more competitive industry.
3. Many technology barriers, once thought to be of concern to a few companies, are common throughout the industry. Overcoming such barriers offers an appropriate focus for technology roadmaps.

NEMI was first motivated in the late 1980s by the U.S. Government. After a few years, industry assumed leadership and provided the funds for NEMI. The history of NEMI suggests that the following lessons should be considered by decision-makers in the compound semiconductor industry:

1. Have discussions with senior managers in industry to obtain their support.
2. Hold preliminary workshops to discuss key issues, identify common interests, and learn about the capabilities of participants.
3. Work from a “virtual product” as a basis for bringing all stakeholders together for planning. The NEMI participants used the personal digital assistant (PDA) as a “virtual product” for providing a focus to their planning efforts. We are proposing here to use delivering wirelessly and in real-time very high quality digital data, such as digital video, as a candidate for providing a focus to the proposed ITRCS.
4. The challenge is to have a large enough effort to be effective, but still focussed enough to have measurable progress.

## 8. Implications for Compound Semiconductors

Si CMOS trends and lessons learned from the ITRS and NEMI histories suggest that compound semiconductor planning needs to be strengthened for commercial and consumer applications. Compound semiconductor planning should consider the following:

1. Smarter investments in compound semiconductors require continuous planning.
2. Planning for compound semiconductors should be international.
3. Because compound semiconductor technologies are very diverse, a focus is needed.
4. Equivalents to Moore’s Law exist for compound semiconductors.
5. Some major markets in III-V compound semiconductors are challenged by Si CMOS, BiCMOS, and SiGe, for example, RFICs.
6. Identify technology challenges for selected applications of compound semiconductors—perhaps, wireless digital video communication networks.

## 9. Action and Impact of an ITRCS

### 9.1 Action Items for an ITRCS

Making progress towards an international technology roadmap for compound semiconductors includes the following steps:

1. Assess which applications are most amenable for developing and supporting an enhanced global infrastructure. Are there areas where companies consider it in their long-term financial interests to share intellectual property and capital?
2. Identify which organizations will sponsor the development and maintenance of the proposed ITRCS and speak for that portion of the compound semiconductor industry.
3. Perform economic assessments of the benefits and costs associated with developing roadmaps and without developing roadmaps for those few selected applications.
4. Convene workshops that bring together scientists, engineers, and managers concerned with strengthening the compound semiconductor infrastructure.
5. Identify the key system applications and technical and commercial problem areas (for example, cost reduction for epi-layer fabrication, millimeter-wave circuits, thermal management, and packaging and mounting of radio frequency devices) and the technology performance gaps between what is or will be available and what the intended market application requires.
6. Rank the gaps in item 5 by perceived technical difficulty and by market priorities.

### 9.2 Impact-Metrics for Assessing Success of an ITRCS

Methods for measuring the impact that the roadmap is having are more elusive. Meaningful metrics applied before the formation of an ITRCS are very difficult to define. As with R&D organizations, the lagging-indicators appear more reliable. Several metrics which indicate leverage and buy-in from the technical and manufacturing communities include:

1. Determine whether the compound semiconductor infrastructure that results from developing and maintaining a roadmap enables manufacturers to go, for example, from costs, yields, reliabilities, and bit-error-rates in a system to acceptable tolerances in processing parameters such as composition, thickness, and doping density.
2. Determine whether sufficient knowledge exists to determine how the above examples of tolerances in processing parameters vary with time and with market application.
3. Determine the extent to which manufacturing yield enhancements are related to the allocation of corporate resources based on ITRCS guidelines.
4. Assess whether actual growth in compound semiconductor markets is greater than the growth projected today or predicted from trends based on historical performance without an ITRCS. For example, it is recognized that the ITRS has accelerated the pace of the silicon CMOS IC industry.
5. Measure the investment that industrial partners commit to roadmap workshops and activities. These are customers’ confirmations based on resources that could be used elsewhere.
6. Document the number of times the roadmap is cited by companies, universities, and governments as a function of time. A strong upward trend signifies acceptance of the roadmap and acknowledgment of its value.

## 10. Digital Video—A Driver for Compound Semiconductors

### 10.1 “Virtual Product” Example

Wireless, real-time digital video (WRTDV) systems (for example, mobile videophones or electronic notebooks) could be used as a “virtual product” in the NEMI-sense to provide the necessary focus for the proposed ITRCS. Digital video in the context of this paper is much broader than its usual meaning. Digital video, as used here, means very high quality and very high bit rates of digital data or information (video, audio, computer, and other forms of digital information) that are processed in real-time. Such systems contain numerous components that span several bands in the electromagnetic wave spectrum; involve microelectronics, optoelectronics, microwave, microelectromechnical (MEMS), and micro-optoelectronics mechanical (MOEMS) systems; require advanced analog-to-digital converters (ADC), digital-to-analog converters (DAC), digital signal processing (DSP), and optoelectronic integrated circuits (OEICs); incorporate transmitters/sources and receivers/detectors; and depend on unique displays, cameras, and sensors.

Technology gaps should be ranked, if possible, according to the perceived technical difficulty and market or economic opportunity. Examples of technology gaps include:

1. miniaturization of microwave filters;
2. low power and very linear microwave amplifiers;
3. circuits with large numbers of HBTs and RTDs for high-speed and high-bit resolution ADCs;
4. reducing timing uncertainties in ADCs;
5. long-lived, continuous semiconductor lasers; and
6. smart, adaptive error correcting circuits for receivers.

### 10.2 Technical Challenges for Digital Video Communications Networks—Opportunities for III-V Compound Semiconductors

The amount of digital signal processing in television receivers is increasing dramatically [17]. Going from analog to digital data as soon as possible brings economic and technical benefits. This is particularly the case for wireless, real-time digital video. Most information originates as analog signals. Digital signal processing is much more versatile and cheaper. Also, digital hardware usually is smaller and uses less power than analog hardware.

In the last few years, major conferences on microelectronics, such as IEDM and GaAs MANTECH, had keynote speakers who discussed the system performance demands that digital video places on microelectronics. The requirements on Si CMOS integrated circuits (IC) for commercial DV [18] are greater and more challenging than some of the performance goals for Si CMOS ICs given in the ITRS [3] and NTRS[19]. One perspective on the technical challenges faced by Si CMOS for DV may be obtained by comparing performance parameters given in Figs. 2, 5, 7, and 9 and Table 1 in Ref. [18] with the corresponding parameters given in Tables 14 and 15 in Ref. [19]. These comparisons show that the digital video industry needs today greater data rates, operations per second, densities of devices, storage capacities, and the like, and that the ITRS goals are not likely to provide such needed performance for many years. For example, designers of HDTV systems would prefer to use high density, low-priced DRAMs instead of magnetic memory with its problem-prone mechanical parts. Storing one hour of HDTV that is compressed by MPEG-2 (MP@HL) requires about 90 Gbit of memory. But, according to the ITRS [3], and only if solutions are discovered for ultra-thin dielectrics, 90 Gbit of DRAM for an HDTV SOC are not expected to be available until about 2009. Also, designers of HDTV cameras would like to have about 500 transistors per pixel in mega-pixel CCDs for image stabilization on the CCD chip itself. That level of integration is not expected to be available until about 2007 and only if overlay and critical dimension control issues are solved. Such challenges, especially those at LMDS frequencies for which mainstream Si CMOS is not expected to be adequate, offer economic opportunities for compound semiconductors and could be the basis of an ITRCS.

The adoption of HDTV and enhanced digital services will exacerbate spectrum crowding and increase the public’s awareness of today’s technical limitations of the hardware and software that are used to deal with multipath and error correction in receivers. Spectrum crowding, multipath, and the need for smart-adaptive error correction combine to make receiving digital video signals difficult, particularly for electronic notebooks and mobile videophones such as the prototypes announced recently [20].

Spectrum crowding may be alleviated by either adding more channels, which means usually higher operating frequencies, or by designing power amplifiers that are very linear. Both going to higher frequencies and using very linear circuits favor III-V compound semiconductors over group IV semiconductors. Economics and technical performance will determine the respective roles for HBTs and MESFETs in alleviating spectrum crowding. Technical performance includes such specifications as power efficiency, linearity, circuit complexity, die size, feature size, and reliability (error rates and lifetimes to failure). Assessments of operating frequency above 10 GHz versus linewidth for MESFET technologies would assist the III-V industry in making its investment decisions. Again, these are all candidate topics for an ITRCS.

Multipath occurs when a broadcast signal is reflected from buildings and other objects, which are either stationary or moving, located between the transmitter and receiver. In the digital domain, multipath manifests itself by the image freezing, breaking up in places into coarse tile patterns, or completely dropping out. Adequately dealing with multipath requires considering both the antenna and receiver as one system. Optimizing the antenna-receiver system with increased use of III-Vs is another ITRCS opportunity.

Spectrum crowding and multipath are two key issues that enter ongoing global discussions, evaluations, and even debates on which modulation/demodulation method from among 8-VSB (U.S.), COFDM (Europe), and modified COFDM (Japan) is the optimum one for acceptable HDTV reception. This debate, the public’s acceptance of HDTV receivers and enhanced digital services, and how well ADCs perform are all interrelated. The ability of error correction hardware and software to handle adequately spectrum crowding (adjacent channel rejection) and multipath depends, in part, on the real-time DSP headroom enabled by the ADC.

### 10.3 Boundary Limit for ADCs—A Moore’s Law

The data in Fig. 1 of Ref. [21] show a significant challenge for all semiconductors—III-Vs, Si, and SiGe; namely, ADCs may not be able to deliver the high quality digital data (video, audio, computer, and multimedia) for market acceptance. Empirical data on the number of bits (resolution) vs. sampling rates show that a boundary—the solid line in Figure 1 of Ref. [21]—exists in the performance of ADCs. It is moving at a given sampling frequency towards higher bit resolutions at the rate of about 1.5 bit every 8 years or so. This motion is too slow for those communication markets that depend on high performance. The ADC performance boundary limits the headroom for which DSPs can handle adequately in real-time the error correcting that are needed due to multipath and adjacent channel interference. For example, in one prototype videophone [20] that uses W-CDMA, the bit rate drops from 2 Mbit/s when stationary to 0.4 Mbit/s when walking. The role that ADC limitations play in decreased bit rates as the speed of mobile receivers increases could be a topic for an ITRCS.

It is conjectured in Ref. [21] that aperture jitter is the main cause of the ADC boundary limit. Emerging technologies based on circuits that contain high counts of InP HBTs, HEMTs, and RTDs have been proposed as a way to move the ADC boundary more quickly. Developing such high-count circuits, understanding better ADC physics, and determining quantitatively the effects on materials processing on ADC performance could all be parts of an ITRCS.

## 11. Proposed Actions—The Next Steps

The two next major steps involve economic assessments and workshops.

### 11.1 Economic Assessments

1. Perform economic assessments for selected segments of the III-V compound semiconductor industry using present technology roadmaps; compare these assessments with estimated economic assessments using an ITRCS similar to the ITRS in scope, namely, an ITRCS with features analogous to items 1 and 2 in Sec. 5; and determine the extent to which the benefits of consensus-based planning outweigh the costs associated with such planning. We know that the benefits of the ITRS and the NTRS outweigh their costs. We also would expect this to be the case for III-V compound semiconductors.
2. Determine economic advantages and disadvantages of future market scenarios. For example, assessments could include determining 1) the costs and benefits of microwave front-end technologies based on III-V compound semiconductors versus those based on Si CMOS compatible technologies; and 2) the economic advantages and disadvantages of increasing the performance of ADCs through the use of III-V compound semiconductors.
3. Collect data as a function of time on the ratio of Si CMOS incompatible content to Si CMOS compatible content in product categories. Such data will quantify the extent to which III-V compound semiconductors have lost market share to Si and SiGe based technologies that are compatible with Si CMOS processing. It would be better if the manufacturers themselves provided such data in a cooperative manner than if third parties used tear-down analyses to obtain these data. Tear-down analyses tend to be burdensome and may not be effective for determining how content ratios vary with time.
4. Gather market sizes, specifications, and acceptance criteria for starting materials (for example, sources of group III and V elements, GaAs wafers, and the like).

### 11.2 Workshops

1. Identify key subsystems and technology performance gaps between what is available today and what will be needed, for example, to make WRTDV a reality.
2. Rank technology gaps according to perceived technical difficulty; illustrative examples may include miniaturization of microwave filters, low power and very linear amplifiers, circuits with large numbers of HBTs and/or RTDs for high speed and high bit resolution ADCs, increased resolution and reduced timing uncertainties in ADCs, and the like.
3. Rank the above technology gaps by market priorities and economic potential.
4. Combine items 2 and 3 to guide investments.

## 12. Conclusions

To deliver its full potential and to ensure its continued success, the III-V compound semiconductor industry needs improved global cooperation from everyone (industry, academia, and governments) to address the tough technical challenges on the road ahead [22]. The successful ITRS for Si CMOS could serve as a template for the III-V compound semiconductor industry. International consensus-based planning offers a way to determine priorities in investing funds to support pre-competitive R&D that will remove technology gaps between what is available and what the markets require. Using these internationally determined technical needs, regional/local decision-makers may then select those needs for which their resources will be used to provide solutions.

We have established a web-site at http://www.eeel.nist.gov/812/itrcs.html for collecting and discussing ideas, comments, and questions relating to the possible creation of ITRCS. Your thoughts are welcome, and can be posted at this site. The compound semiconductor industry at times seems quite fragmented, and some segments of our industry have a certain entrepreneurial character that may seem incompatible with the concept of international consensus-based planning. It is therefore essential to consider the words of Dr. Avtar Oberai, formerly from IBM and a founding director of SEMATECH: “No one is big enough to drive the totality of the infrastructure and pre-competitive investments on their own.” Oberai was a key player in bringing about collaborative planning for the silicon industry. The compound semiconductor industry has much to learn from his experiences and from others in the silicon industry.

## Footnotes

1 Certain commercial equipment, instruments, or materials are identified in this paper to foster understanding. Such identification does not imply recommendation or endorsement by the National Institute of Standards and Technology, nor does it imply that the materials or equipment identified are necessarily the best available for the purpose.

## Glossary

8-VSB: 8-level vestigial side band
ADC: analog-to-digital converter
BiCMOS: bipolar complementary metal-oxide-semiconductor
CCD: charge coupled device
CDMA: code division multiple access
CMOS: complementary metal-oxide-semiconductor
COFDM: coded frequency division multiplexing
DAC: digital-to-analog converter
DRAM: dynamic random access memory
DSP: digital signal processing
DV: digital video
FET: field effect transistor
GaAs MANTECH GaAs: Manufacturing Technology
HBT: heterojunction bipolar transistor
HDTV: high definition television
HEMT: high electron mobility transistor
IC: integrated circuit
IEDM: International Electron Devices Meeting
ITRCS: International Technology Roadmap for Compound Semiconductors
ITRS: International Technology Roadmap for Semiconductors
LMDS: local multipoint distribution service
MEL-ARI-OPTO: Microelectronics Advanced Research Initiative Optoelectronics
MEMS: microelectromechnical system
MESFET: metal semiconductor field effect transistor
MICROTECH2000: Microelectronics Technology 2000
MOEMS: micro-optoelectronics mechanical system
MPEG: Motion Picture Experts Group
MP@HL: main profile at high level
NEMI: National Electronics Manufacturing Initiative
NTRS: National Technology Roadmap for Semiconductors
OEIC: optoelectronic integrated circuits
OIDA: Optoelectronics Industry Development Association
OITDA: Optoelectronics Industry and Technology Development Association
PDA: personal digital assistant
R&D: research and development
rf: radio frequency
RFIC: radio frequency integrated circuit
RTD: resonant tunneling diode
SEMATECH: Semiconductor Manufacturing Technology
SIA: Semiconductor Industry Association
SOC: system on a chip
SRC: Semiconductor Research Corporation
W-CDMA: wide-band code division multiple access
WRTDV: wireless real-time digital video
WWW: world wide web

## References

[1] Bennett HS (1996). Conference report on planning for compound semiconductor technology. J Res Natl Inst Stand Technol.

[2] Bennett HS (1999). Viewpoint: Technology roadmaps for compound semiconductors—Knowledge-based investments for the next century. IEEE Spectrum.

[3] Semiconductor Industry Association International Technology Roadmap for Semiconductors.

[4] Luryi S, Xu J, Zaslavsky A (1999). Future Trends in Microelectronics: The Road Ahead.

[5] (1999). International Technology Roadmap for Semiconductors.

[6] de Solla Price D (1986). Little Science, Big Science And Beyond.

[7] Metzger RA (1998). Silicon Update. Compound Semiconductor.

[8] Ning T (1999). With SiGe, Who Needs GaAs?. Distinguished Lecture.

[9] (1998). Technology Business.

[10] Chyan YF, Chaudhry S, Ma Y, Chen A, Carroll MS, Nagy WJ, Becerro J, Lee JL, Iannuzzi MP, Lee KH A very high performance, epi-free, and manufacturable 3.3 V 0.35 μm BiCMOS technology for wireless applications.

[11] Metzer RA (1998). BiCMOS for Communications. Compound Semiconductor.

[12] Komiya H The 300mm technology: current Status and future prospect.

[13] National Electronics Manufacturing Technology Roadmaps.

[14] MEL-ARI Optoelectronics Technology Roadmap.

[15] Optoelectronics Technology Roadmap.

[16] Hirahara K, Fujii T, Ishida K, Ishihara S (1998). Optical communications technology roadmap. IEICE Trans Electron.

[17] A. Thygessen, remarks made during the discussion panel, PC/TV – TV/PC: Where Are We Headed and Who Is Winning?.

[18] Sasaki H Multimedia: future and impact for semiconductor technology.

[19] Semiconductor Industry Association (1997). The National Technology Roadmap for Semiconductors: Technology Needs.

[20] Videomaker Magazine (1999). Wireless Videophones are Coming.

[21] (1999). ADC survey and analysis. IEEE Communications Magazine.

[22] Pickering S (1999). Finding the Road Ahead, in an editorial. III-Vs Review.

